# Sulfation of a FLAG tag mediated by SLC35B2 and TPST2 affects antibody recognition

**DOI:** 10.1371/journal.pone.0250805

**Published:** 2021-05-05

**Authors:** Xin-Yu Guo, Xiao-Dong Gao, Morihisa Fujita

**Affiliations:** Key Laboratory of Carbohydrate Chemistry and Biotechnology, Ministry of Education, School of Biotechnology, Jiangnan University, Wuxi, Jiangsu, China; Hirosaki University Graduate School of Medicine, JAPAN

## Abstract

A FLAG tag consisting of DYKDDDDK is an epitope tag that is frequently and widely used to detect recombinant proteins of interest. In this study, we performed a CRISPR-based genetic screening to identify factors involved in the detection of a FLAG-tagged misfolded model protein at the cell surface. In the screening, *SLC35B2*, which encodes 3’-phosphoadenosine-5’-phosphosulfate transporter 1, was identified as the candidate gene. The detection of FLAG-tagged misfolded proteins at the cell surface was significantly increased in *SLC35B2*-knockout cells. Furthermore, protein tyrosine sulfation mediated by tyrosyl-protein sulfotransferase 2 (TPST2) suppressed FLAG-tagged protein detection. Localization analysis of the FLAG-tagged misfolded proteins confirmed that defects in tyrosine sulfation are only responsible for enhancing anti-FLAG staining on the plasma membrane but not inducing the localization change of misfolded proteins on the plasma membrane. These results suggest that a FLAG tag on the misfolded protein would be sulfated, causing a reduced detection by the M2 anti-FLAG antibody. Attention should be required when quantifying the FLAG-tagged proteins in the secretory pathway.

## Introduction

Protein tags are peptide sequences that can be grafted onto a target recombinant protein. Protein tags can be removed by chemical agents or by enzymatic reactions. A FLAG tag is a commonly and widely used protein tag that could be added either to the N-terminus, C-terminus or intermediate regions of the targeted protein. The amino acid sequence motif of the FLAG-tag is DYKDDDDK, which contains an enterokinase cleavage site. Some types of commercially available antibodies (e.g., M1/4E11) recognize the epitope only when it is present at the N-terminus. However, other available antibodies (e.g., M2) show no position sensitivity. Previously, several studies have reported that FLAG is not a good protein tag because a tyrosine residue on the FLAG-tag can be sulfated [1, 2]. Some commercial antibodies, for example, M2, seem sensitive to the sulfation state of the FLAG tag [2, 3].

Sulfation is a post-translational modification that occurs in the *trans*-Golgi network (TGN) [4]. Both proteins and glycans can be modified with sulfates. SLC35B2 is 3’-phosphoadenosine-5’-phosphosulfate (PAPS) transporter 1, which transports the sulfate donor PAPS from the cytosol to the TGN. For protein sulfation, sulfate can be modified to a tyrosine residue on proteins, which is mediated by tyrosyl-protein sulfotransferases 1 and 2 (TPST1&2) [5, 6]. A tyrosine residue surrounded by negatively charged amino acids (e.g., glutamic acid and aspartic acid) on the peptide is recognized as a substrate for the enzymes [7]. Based on this rule, a prediction software for tyrosine sulfation was developed (https://web.expasy.org/sulfinator/) [8]. In most cases, tyrosine sulfation does not affect target protein stability and localization, whereas it changes protein function and antibody affinity [1, 9]. CCR5, which acts as a HIV co-receptor, receives tyrosine sulfation, which is essential for HIV infection [9].

Glycosylphosphatidylinositol (GPI) is a glycolipid utilized for membrane tethering of plasma membrane proteins [10–12]. GPI modification of proteins is carried out in the endoplasmic reticulum (ER). In the ER, there are quality control systems that monitor the folding status of proteins [13]. When the proteins fail to fold properly, they are recognized as misfolded proteins and degraded through the ER-associated degradation (ERAD) pathway [14]. However, fractions of misfolded GPI-anchored proteins (GPI-APs) are not degraded through ERAD, probably due to the presence of GPI anchors [15–17]. In mammalian cells, although misfolded GPI-APs are retained in the ER, they are rapidly released into the secretory pathway once acute ER stress was given [17]. Even under normal conditions, a small fraction of misfolded prion, CD59, and CD55 are constantly transported to the plasma membrane [18, 19].

In this study, we performed genome-wide CRISPR-Cas9 screening to identify factors involved in the surface expression of misfolded FLAG-tagged GPI-APs. Mutant cells whose surface was highly stained with the anti-FLAG antibody were enriched, and knockout genes were determined. *SLC35B2* was identified as the top gene in the screening. SLC35B2-KO and TPST1&TPST2-DKO cells showed higher anti-FLAG staining, presuming that protein tyrosine sulfation changes the localization of the misfolded FLAG-tagged GPI-AP to the cell surface. However, after we changed the FLAG tag to the MYC tag, the phenotype disappeared in SLC35B2-KO cells. Under confocal microscopy, we found that the majority of EGFP-FLAG-CD55 (C81A) still localized in the ER, not on the plasma membrane, similar to wild-type (WT) cells. Our results indicate that knocking out *SLC35B2* or *TPST1* and *TPST2* does not induce misfolded GPI-APs to migrate to the plasma membrane. Rather, the FLAG tag on misfolded GPI-APs might be sulfated, causing the low reactivity of the anti-FLAG antibody.

## Materials and methods

### Cells and culture

HEK293 cells and their KO derivative cells were cultured in Dulbecco’s modified Eagle medium (DMEM) containing 10% (vol/vol) FCS (Biological Industries). Streptomycin/penicillin (1 μg/ml) was used where necessary. Cells were maintained at 37°C and 5% CO_2_ in a humidified atmosphere. pPB-FRT-PGKp-BSD-mEGFP-FLAG-CD55 (C81A) and pCMV-hyPBase [20] were cotransfected into HEK293 WT cells and selected with 10 μg/ml blasticidin for stable expression. A single clone of HEK293 cells stably expressing mEGFP-FLAG-CD55 (C81A) was isolated by limiting dilution and was used for a genetic screen. For CRISPR knockout screening using the GeCKO library [21], 1 μg/ml puromycin was used to select cells that were infected by lentivirus. SLC35B2-KO cells stably expressing SLC35B2 and TPST1&2-DKO cells stably expressing TPST1 and/or TPST2 were established by retrovirus-based vector infection, followed by selection with 400 μg/ml hygromycin B.

### Antibodies and materials

Mouse anti-FLAG (M2; Sigma and HT201-01; Transgen), anti-calnexin (M178-3; MBL), anti-c-MYC (9E10; Santa Cruz), and rabbit anti-FLAG (20543-1-AP; Proteintech) were used as the primary antibodies. Phycoerythrin (PE)-conjugated goat anti-mouse IgG (eBioscience), PE-conjugated donkey anti-rabbit IgG (eBioscience), and Alexa Fluor 555-conjugated F(ab’)2-goat anti-mouse IgG (H+L) cross-adsorbed secondary antibodies (Thermo Fisher Scientific) were used as the secondary antibodies. NaClO_3_ (403016; Sigma) was used for drug treatments.

### Plasmids

For the CRISPR-Cas9 systems to knock out target genes, guide RNA sequences were designed by the E-CRISP website [22] (http://www.e-crisp.org/E-CRISP/), and the designed DNA fragments were ligated into *Bpi*I-digested pX330-EGFP. All the primers used in this study are listed in S1 Table. The *SLC35B2*, *TPST1*, and *TPST2* cDNA fragments were amplified from human cDNA and cloned into the retroviral vector pLIB2-Hyg to generate pLIB2-Hyg-SLC35B2, TPST1 or TPST2. The DNA fragment coding mEGFP-FLAG-CD55 was digested with *Eco*RI and *Not*I and cloned into pPB-FRT-PGKp-BSD to generate the pPB-FRT-PGKp-BSD-mEGFP-FLAG-CD55 plasmids as previously described [20]. The misfolded mutant of CD55 (C81A) was constructed by site-direct mutation. pPB-FRT-PGKp-BSD-mEGFP-MYC-CD55 WT or C81A was constructed by infusion. A 3×FLAG-CD55(C81A) DNA fragment was amplified from pPB-FRT-EGFP-FLAG-CD55(C81A) by PCR. The 3×FLAG-CD55(C81A) fragment was then digested with *Sal*I and *Not*I and cloned into pPB-FRT-EGFP, generating pPB-FRT-EGFP-3FLAG-CD55(C81A).

### Establishment of knockout cell lines

To generate gene knockout cell lines, HEK293 cells stably expressing EGFP-FLAG-CD55 (C81A) or HEK293 cells were transiently transfected with pX330-EGFP plasmids bearing the target sequences. Three days after transfection, cells with EGFP were sorted by a S3e cell sorter (Bio-Rad). The collected cells were cultured for 8 days and subjected to limiting dilution to obtain clonal KO cells. A clone that had no wild-type allele was picked up. DNA sequences were analyzed by the Sanger method. SLC35B2-KO cell lines were established from both HEK293 cells and HEK293 cells stably expressing EGFP-FLAG-CD55 (C81A). The details of KO cell line are listed in S2 Table.

### A genetic screen using GeCKO library

HEK293T cells (6.5 × 10^6^ cells per 10-cm dish) were seeded in 2 × 10-cm dishes and incubated for 12–16 h. The plasmid mixture contained pMD2.G (4.5 μg), psPAX2 (6.7 μg), GeCKO-lib A (4.5 μg), GeCKO-lib B (4.5 μg) (pMD2. G: psPAX2: GeCKO-lib A: GeCKO-lib B = 1:1.5:1:1) were transfected into HEK293T cells. Twelve hours after transfection, the medium was replaced with prewarmed DMEM containing 10% FBS. After 24, 48, and 72 h of changing the medium, the supernatant containing lentiviral vectors was collected, combined, and stocked at 4°C. The lentiviral supernatant was filtered through a 0.45 μm PVDF filter. After filtration, the supernatant was quickly frozen in liquid nitrogen and stored at -80°C until use.

For screening, HEK293 cells stably expressing EGFP-FLAG-CD55 (C81A) were used as parental cells. Cells (3.5–4 × 10^6^ cells per 15-cm dish; total 6 dishes) were seeded and cultured for 36 h. Then, 500 μl of lentiviral supernatant was added into each 15-cm dish (MOI was precalculated as 0.3) and incubated for a half day, and prewarmed fresh medium was changed. After 12 h of culture, the cells were harvested and selected with puromycin. Cells were maintained in medium containing puromycin and cultured in 12 × 15-cm dishes. Two dishes were directly harvested for the non-screening control, the other 10 dishes were stained with the anti-FLAG antibody and PE-conjugated goat anti-mouse IgG, and cells stained with the anti-FLAG antibody were sorted by the S3e cell sorter. The cells that were highly stained with the anti-FLAG antibody were enriched three times.

### Determination of sgRNA sequences

Genomic DNA was isolated from 3 × 10^7^ cells, either non-selected control or cells after sorting, using the Wizard Genomic DNA Purification Kit (Promega) according to the manufacturer’s protocol. After purification, the genomic DNAs were used as templates for PCR amplification, and GeCKO-F2 (5’- ATCATGCTTAGCTTTATATATCTTGTGGAAAGGACGAAACACC-3’) and GeCKO-R (5’- CCGACTCGGTGCCACTTTTTCAA-3’) were used as forward and reverse primers. After purification, the amplicons were analyzed by Illumina HiSeq. Deep sequencing raw data were processed for sgRNA counting using Python scripts. These sgRNA sequences were mapped to the sequences of the Human GeCKO v2 sgRNA library, and the total number of sgRNA counts was obtained by using the MAGeCK workflow [23]. The robust rank aggregation (RRA) values and p-values were determined using the MAGeCK algorithm.

### Flow cytometric analysis

HEK293 cells were transfected with pME-EGFP-FLAG-CD55 (C81A) by Lipofectamine 2000 (Thermo Fisher Scientific). Thirty-six hours after transfection, cells were treated with 50 mM NaClO_3_ for 36 h. Cells (10^6^) were harvested with 2 mM EDTA diluted in D-PBS. After being washed with PBS, the cells were stained with anti-FLAG (M2; Sigma-Aldrich) (10 μg/ml) for 25 min on ice and then washed two times with cold FACS buffer (PBS containing 1% BSA and 0.1% NaN_3_). Subsequently, the cells were incubated with PE-conjugated goat anti-mouse IgG as the secondary antibody for 25 min on ice, washed two times with FACS buffer and then analyzed with an Accuri C6 instrument (BD). The data were analyzed using FlowJo (BD).

### Immunofluorescence analysis

To detect the subcellular localization of misfolded CD55 (EGFP-FLAG-CD55 (C81A)), HEK293 and its KO derivative cells stably expressing EGFP-FLAG-CD55 (C81A) were used. For expression of mRFP-KDEL, HEK293 cells stably expressing EGFP-FLAG-CD55 (C81A) were transfected with pME-Zeo-mRFP-KDEL. Then, 36 h after transfection, cells were replated onto glass coverslips pretreated with 1% gelatin and cultured for another 2 days. Subsequently, the cells were washed with PBS, fixed and permeabilized with -20°C methanol for 5 min at 4°C before being blocked with PBS containing 5% FCS (blocking buffer) for 1 h. The cells were incubated with mouse anti-calnexin (M178-3; MBL) as the primary antibody diluted in blocking buffer for 1 h. After washing twice with PBS, the cells were incubated with an F(ab’)2-goat anti-mouse IgG (H+L) cross-adsorbed secondary antibody, Alexa Fluor 555 diluted in blocking buffer for 1 h, after which the cells were gently washed with PBS twice. The coverslips were mounted onto slides using a mounting solution containing DAPI for 5 min. The cells were visualized using a confocal microscope (C2si; Nikon) with a CFI Plan Apochromat VC oil objective lens (100× magnification and 1.4 NA).

## Results

### Genome-wide CRISPR-Cas9 knockout screening identified mutations in sulfo-modification that increase anti-FLAG staining on the plasma membrane

**Fig 1:**
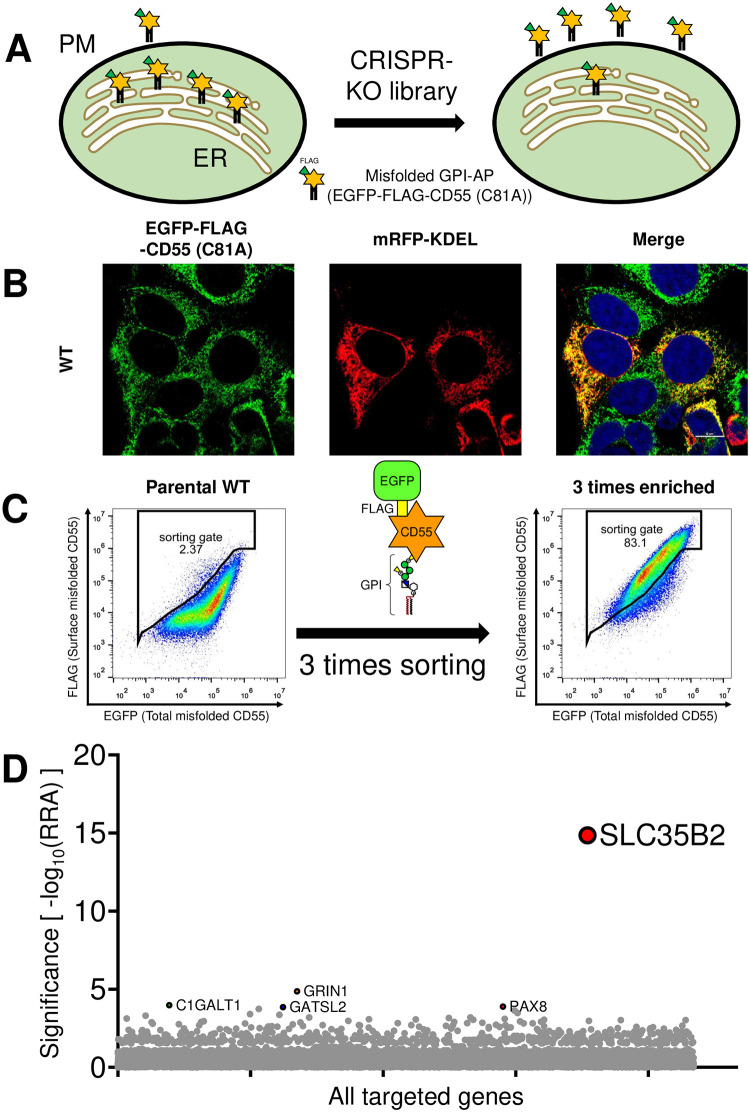
A CRISPR-based genetic screen to identify genes involved in the surface detection of FLAG-tagged misfolded GPI-APs. A. Scheme of CRISPR screening. Under normal conditions, the majority of misfolded GPI-APs are retained in the ER, with only a small fraction of them on the plasma membrane (PM) (left). After mutagenesis using the CRISPR KO library, cells in which misfolded GPI-APs are expressed on the PM were enriched using the cell sorter (right). For a model misfolded GPI-AP, EGFP-FLAG-CD55 (C81A) was used. A FLAG-tag was utilized for the detection. B. Localization of misfolded EGFP-FLAG-CD55 (C81A). An ER marker, mRFP-KDEL, was transiently transfected into HEK293 WT cells stably expressing EGFP-FLAG-CD55 (C81A). The fluorescence of EGFP was detected to analyze the localization of EGFP-FLAG-CD55 (C81A). Scale bar, 10 μm. C. Flow cytometric analysis of parental HEK293 WT cells stably expressing EGFP-FLAG-CD55 (C81A) (left) and cells after enrichment 3 times using a cell sorter (right). Cells were harvested and stained with the anti-FLAG antibody. x-axis, fluorescence intensity of EGFP in cells (total EGFP-FLAG-CD55 (C81A)); y-axis, fluorescence intensity of cells stained with the anti-FLAG antibody followed by PE-conjugated anti-mouse IgG (surface EGFP-FLAG-CD55 (C81A)). D. Gene scores in unsorted HEK293 cells stably expressing EGFP-FLAG-CD55 (C81A) cells versus cells enriched 3 times by cell sorting. Five top-ranking protein-coding genes are shown. Bubble size shows the number of active sgRNAs per gene.

Under steady states, the majority of misfolded GPI-APs are retained in the ER, but fractions of them constantly reach the plasma membrane before going to the lysosome for degradation. Under the microscope, the majority of misfolded prions (PrP*) are detected in the ER [17], but proportions of PrP* [18], CD55 (C81A) and CD59 (C94S) [19] are detected on the plasma membrane. Based on the data, we tried to identify factors that are involved in the retention of misfolded GPI-APs in the ER using the genome-scale CRISPR-Cas9 knockout (GeCKO) library [21] (Fig 1A). HEK293 cells stably expressing EGFP-FLAG-CD55 (C81A) were used as the parental cells. EGFP-FLAG-CD55 (C81A) was mainly localized in the ER (Fig 1B) but was also weakly detected on the plasma membrane by the anti-FLAG antibody (Fig 1C, left). The fluorescence derived from EGFP indicates the total level of EGFP-FLAG-CD55 (C81A), while the anti-FLAG staining level indicates the cell surface level. After mutagenesis by the GeCKO library, cells that were highly stained with the anti-FLAG antibody were enriched 3 times (Fig 1C, right). After extraction of genomic DNA from enriched cells and non-selected cells, target guide RNAs in the cells were determined by deep sequencing. *SLC35B2*, which encodes 3’-phosphoadenosine-5’-phosphosulfate transporter 1 [24, 25], was highly enriched in the screening (Fig 1D). 3’-Phosphoadenosine-5’-phosphosulfate (PAPS) is a substrate utilized for various sulfo-modifications. The function of SLC35B2 is to transport PAPS from the cytosol into the Golgi lumen, where sulfo-modification is carried out. These results suggest that deletion of sulfation in the Golgi increases the detection of EGFP-FLAG-CD55 (C81A) on the cell surface.

**Fig 2:**
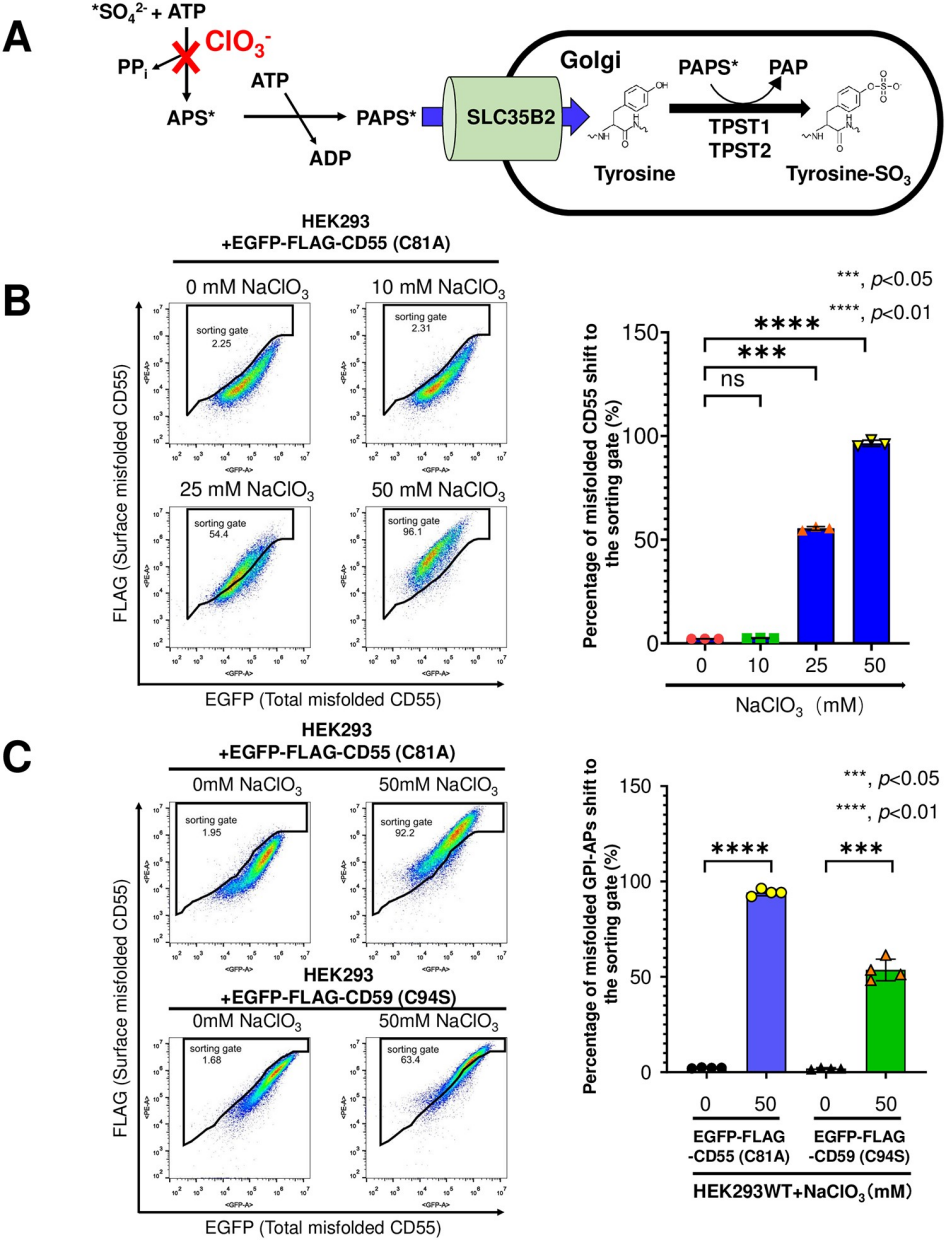
Defects in sulfo-modifications increase anti-FLAG staining on the cell surface. A. SLC35B2 is the major transporter of PAPS, which is a substrate donor of sulfo-modifications, from the cytosol to the Golgi. TPST1 and TPST2 act as tyrosyl protein sulfotransferases that add sulfate to the tyrosine residues of proteins. The PAPS biosynthesis is inhibited by ClO_3_^-^. *, a sulfate molecule utilized for sulfation. B. HEK293 WT cells stably expressing EGFP-FLAG-CD55 (C81A) were treated with 0 mM, 10 mM, 25 mM, and 50 mM NaClO_3_ for 48 h. The surface expression of EGFP-FLAG-CD55 (C81A) was analyzed by flow cytometry as described in Fig 1C (left). The percentages of the positive gate are presented as the means ± SD from three independent experiments. P-values (two-tailed, student’s t-test) are shown (right). C. HEK293 WT cells stably expressing EGFP-FLAG-CD55 (C81A) or EGFP-FLAG-CD59 (C94S) were treated with 50 mM NaClO_3_ for 48 h. The surface expression of EGFP-FLAG-CD55 (C81A) and EGFP-FLAG-CD59 (C94S) was analyzed by flow cytometry as described in Fig 1C (left). The percentages of the positive gate are presented as the means ± SD from four independent experiments. P-values (two-tailed, student’s t-test) are shown (right).

To confirm whether sulfation affects the detection of EGFP-FLAG-CD55 (C81A) on the cell surface, cells were treated with NaClO_3_, which is an inhibitor of ATP-sulfurylase and the first enzyme in the biosynthesis pathway of PAPS [26, 27] (Fig 2A). By treating HEK293 cells stably expressing EGFP-FLAG-CD55 (C81A) with different concentrations of NaClO_3_ for 48 h, anti-FLAG staining on the cell surface was increased. A higher concentration of NaClO_3_ increased the staining (Fig 2B). To analyze whether this phenomenon is observed in another misfolded GPI-AP, HEK293 cells stably expressing EGFP-FLAG-CD59 (C94S) were treated with 50 mM NaClO_3_ and stained with the anti-FLAG antibody. Similar to EGFP-FLAG-CD55 (C81A), FLAG staining of EGFP-FLAG-CD59 (C94S) was increased by treatment with NaClO_3_ (Fig 2C).

### TPST2-mediated tyrosine sulfation mainly affects the FLAG staining

**Fig 3:**
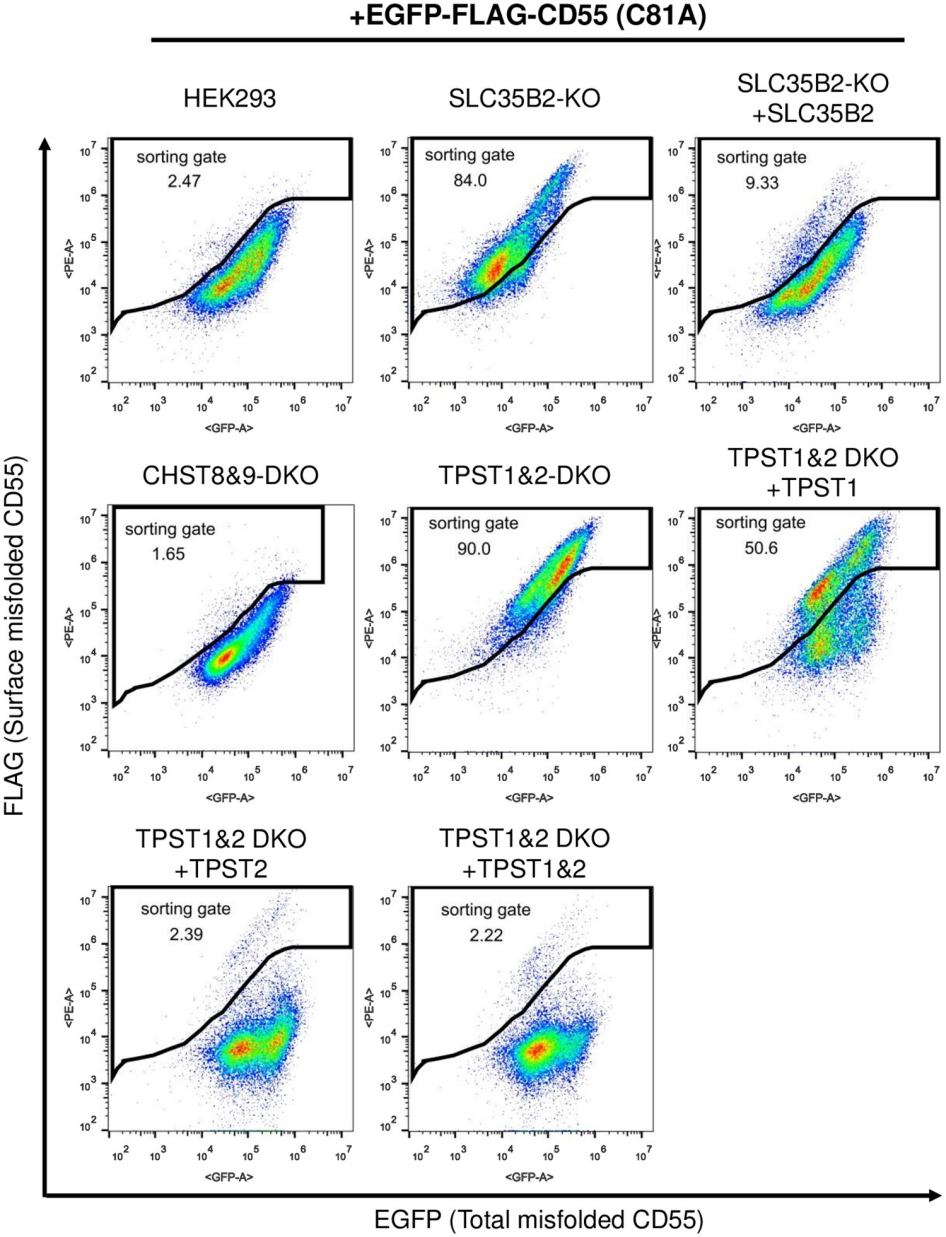
TPST2-mediated tyrosine sulfation mainly affects FLAG staining. Flow cytometric analysis of EGFP-FLAG-CD55 (C81A) in HEK293 WT, SLC35B2-KO, SLC35B2-KO stably expressing *SLC35B2*, TPST1&2-DKO, CHST8&9-DKO, TPST1&2-DKO rescued with *TPST1* and/or *TPST2*. The surface expression of EGFP-FLAG-CD55 (C81A) was analyzed by flow cytometry as described in Fig 1C.

We next knocked out the *SLC35B2* gene in HEK293 cells stably expressing EGFP-FLAG-CD55 (C81A). Surface FLAG staining in *SLC35B2*-knockout (KO) cells was analyzed by flow cytometry. Compared to the parental WT cells, FLAG staining was clearly increased in SLC35B2-KO cells, confirming the results of CRISPR-KO screening (Fig 3). The increased staining in SLC35B2-KO cells was normalized by rescuing *SLC35B2* expression.

In the Golgi, proteins and glycans are sulfated. Protein sulfation occurs on the tyrosine residue of proteins mediated by tyrosyl protein sulfotransferase 1 or 2 (TPST1 or TPST2) [5, 6, 28]. Various glycans, including N-glycans, mucin-type O-glycans, and glycosaminoglycans, receive sulfo-modifications [29], which are mediated by sulfotransferases. For example, CHST8 and CHST9 transfer sulfate to position 4 of N-acetylgalactosamine (GalNAc) residues on N,N’-diacetyl-lactosamine (LacdiNAc) structures in both N-glycans and O-glycans [30, 31]. To clarify which sulfation affects the staining of EGFP-FLAG-CD55 (C81A) on the cell surface, we constructed *TPST1* and *TPST2* double KO (TPST1&2-DKO) and *CHST8* and *CHST9* double KO (CHST8&9-DKO) cells based on HEK293 cells stably expressing EGFP-FLAG-CD55 (C81A). FLAG staining in TPST1&2-DKO cells, but not CHST8&9-DKO cells, was increased, similar to that in SLC35B2-KO cells (Fig 3), suggesting that the removal of sulfation on the tyrosine residue is important for cell surface staining. When TPST1&2-DKO cells were rescued by transfection with *TPST1*, the phenotype was only partially restored. On the other hand, FLAG staining was strongly decreased by the overexpression of *TPST2* (Fig 3), indicating that TPST2 plays a major role in transferring sulfate to the tyrosine residue to show the phenotype.

### Tyrosine sulfation on the FLAG-tag affects anti-FLAG antibody reactivity

**Fig 4:**
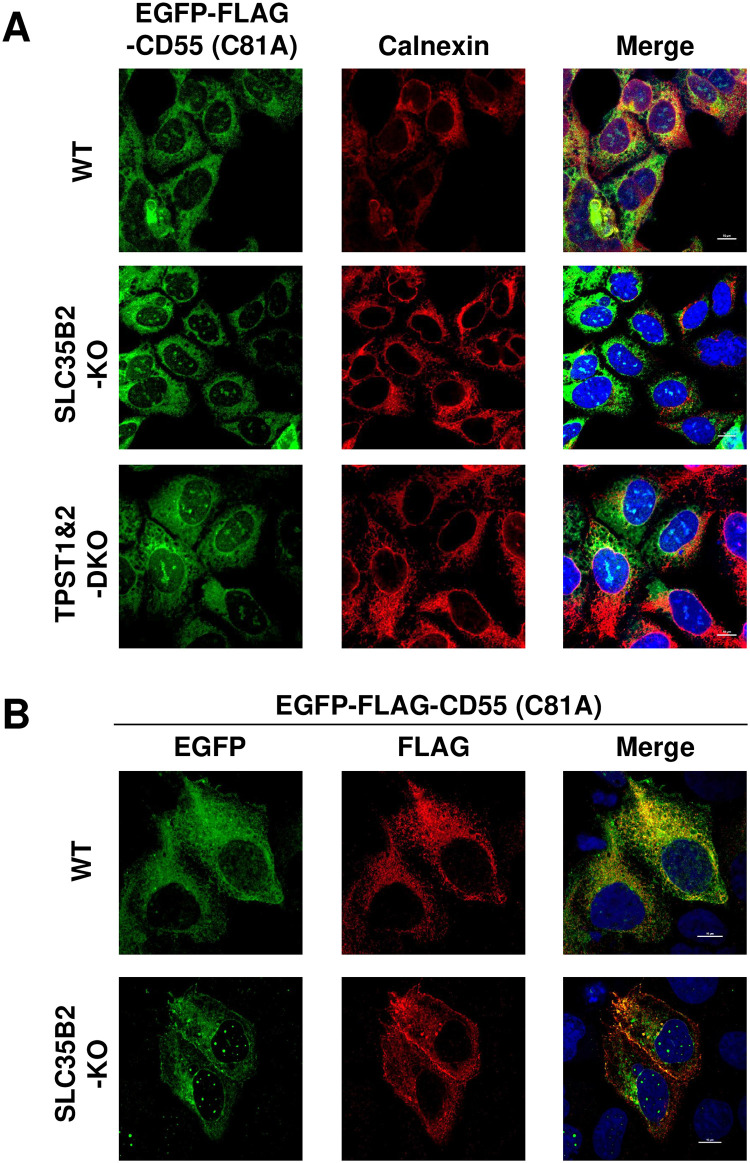
Localization of misfolded GPI-APs are not changed by impairment of tyrosine sulfation. A. Localization of EGFP-FLAG-CD55 (C81A) in HEK293 WT, SLC35B2-KO and TPST1&2-DKO cells. Cells stably expressing EGFP-FLAG-CD55 (C81A) were fixed and stained with an anti-calnexin antibody followed by an anti-mouse Alexa Fluor 555 to analyze ER localization. The fluorescence of EGFP was detected to analyze the localization of EGFP-FLAG-CD55 (C81A). Scale bar, 10 μm. B. EGFP-FLAG-CD55 (C81A) in HEK293WT and SLC35B2-KO was stained by anti-FLAG antibody. Cells were transiently transfected with a plasmid expressing EGFP-FLAG-CD55 (C81A). Three days after transfection, cells were fixed with methanol. Anti-FLAG (Sigma, M2) antibody, followed by an anti-mouse-Alexa Fluor 555, were used for staining. The fluorescence derived from EGFP and FLAG was detected by confocal microscopy. Scale bar, 10 μm.

Our initial purpose of the genetic screening was to identify factors involved in the retention of misfolded GPI-APs in the ER. Since FLAG staining on the cell surface was increased by KO of *SLC35B2* or *TPST1&2*, we next analyzed whether this phenomenon occurred due to changes in the localization of misfolded GPI-APs from the ER to the plasma membrane. The localization of EGFP-FLAG-CD55 (C81A) was detected in SLC35B2-KO cells and TPST1&2-DKO cells using confocal microscopy. Contrary to our expectation, the majority of EGFP signals derived from EGFP-FLAG-CD55 (C81A) were detected in the ER, similar to that in WT cells (Fig 4A). When the cells were stained by M2 anti-FLAG antibody, signals from anti-FLAG antibody were detected in the ER in WT cells (Fig 4B). On the other hand, in SLC35B2-KO cells, fractions of signals from anti-FLAG antibody were detected on the cell surface as well as ER region. EGFP signals on the cell surface were also changed in SLC35B2-KO cells, when the cells were stained with anti-FLAG antibody (Fig 4B). However, it seems to be an artifact caused by crosslinking of EGFP-FLAG-CD55 (C81A) using anti-FLAG antibody, since EGFP signals were dispersed and not obviously detected on the cell surface in both WT and SLC35B2-KO cells without anti-FLAG antibody. The difference in signals between EGFP and FLAG raises the possibility that a tyrosine residue on a FLAG-tag is sulfated, causing a reduction in antibody reactivity.

**Fig 5:**
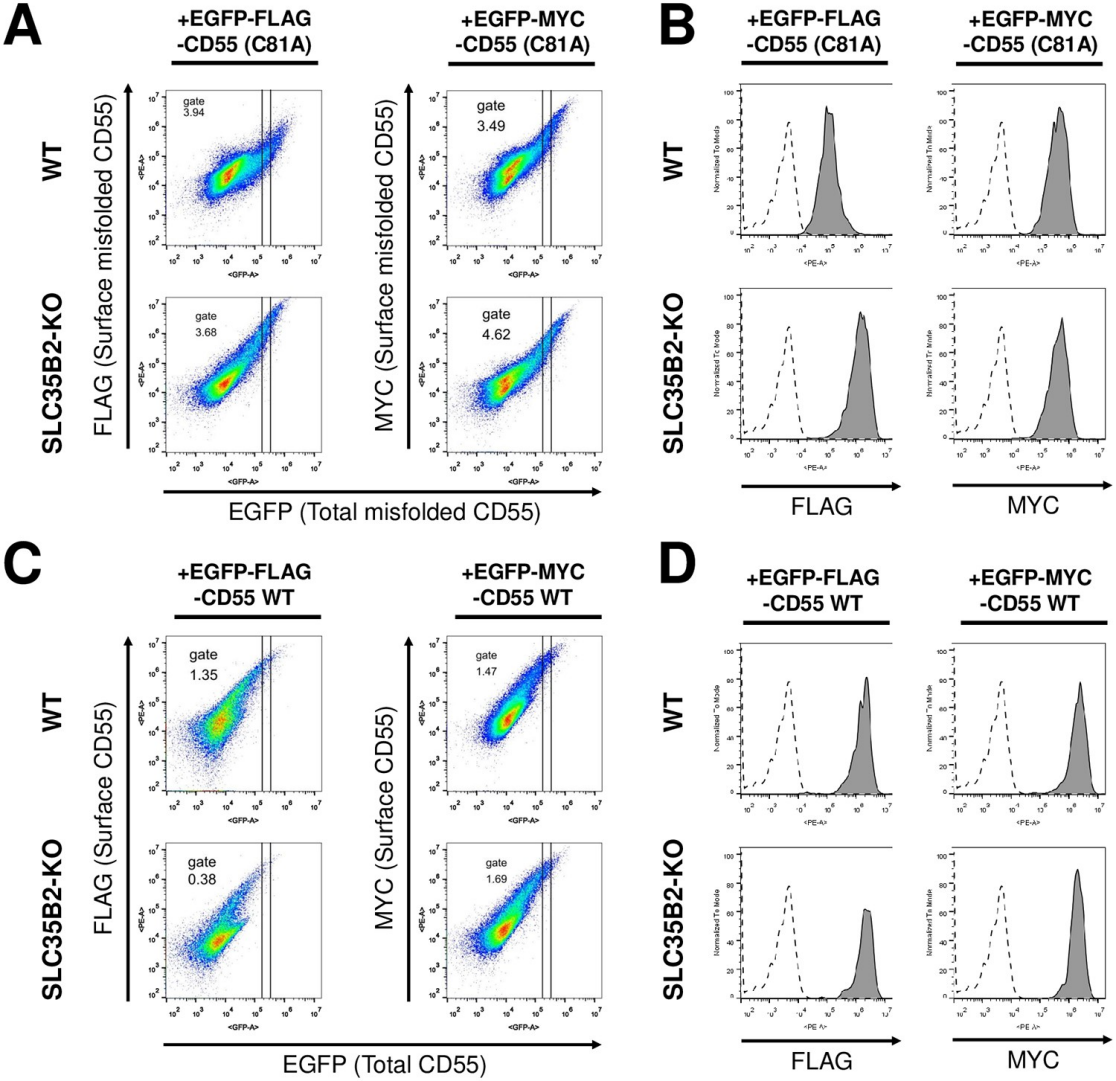
Tyrosine sulfation on the FLAG-tag affects reactivity to the anti-FLAG antibody. A-D. HEK293 WT and SLC35B2-KO cells were transiently transfected with EGFP-FLAG-CD55 (C81A) or EGFP-MYC-CD55 (C81A) (A and B) or EGFP-FLAG-CD55 WT or EGFP-MYC-CD55 WT (C and D). Three days after transfection, cells were harvested and analyzed with anti-FLAG or anti-MYC antibody as described in Fig 1C (A and C). The GFP-positive regions were gated, and the fluorescence intensities of FLAG or MYC staining were analyzed by flow cytometry (B and D). The results are representative of three independent experiments.

To confirm this possibility, a FLAG tag on EGFP-FLAG-CD55 was replaced with a MYC tag. EGFP-FLAG-CD55 (C81A) or EGFP-MYC-CD55 (C81A) was transiently transfected into wild-type and SLC35B2-KO HEK293 cells. In SLC35B2-KO cells expressing EGFP-FLAG-CD55 (C81A), surface staining by the anti-FLAG antibody was increased compared with WT cells (Fig 5A, left, Fig 5B, left). In contrast, MYC staining of SLC35B2-KO cells expressing EGFP-MYC-CD55 (C81A) was not changed compared with that of wild-type cells (Fig 5A, right, Fig 5B, right). We further constructed 3×FLAG tagged CD55 (C81A) (EGFP-3FLAG-CD55 (C81A)) to analyze the staining. Similar to EGFP-FLAG-CD55 (C81A), FLAG staining of EGFP-3FLAG-CD55 (C81A) on the cell surface was increased in SLC35B2-KO cells (S1A Fig). These results suggest that the phenomena observed in SLC35B2-KO cells were specific to cells expressing FLAG-tagged misfolded GPI-APs. Based on the amino acid sequence of the FLAG-tag and previous reports [1, 2], it is reasonable that tyrosine sulfation occurs on the FLAG-tag, which affects antibody reactivity.

In all the experiments in this study, we used the M2 anti-FLAG antibody, which is most widely and frequently used to detect the FLAG-tagged proteins. To check the specificity of the phenomena observed in M2 anti-FLAG antibody, different anti-FLAG antibodies (monoclonal from Transgen (HT201-01) and polyclonal from Proteintech (20543-1-AP)) were used for FACS analysis (S1B Fig). The monoclonal anti-FLAG antibody from Transgen showed no obvious difference between WT and SLC35B2-KO cells. Compared with the staining by M2 anti-FLAG, FLAG was highly stained regardless in WT and SLC35B2-KO cells. On the other hand, the polyclonal antibody from Proteintech showed similar staining patterns with M2 antibody in WT and SLC35B2-KO cells. These results suggest that some antibody against FLAG-tag is sensitive to the tyrosine-modification on the epitope.

We finally analyzed whether the FLAG modification specifically occurred in misfolded GPI-APs. EGFP-FLAG-CD55 (WT) and EGFP-MYC-CD55 (WT) were transiently expressed in wild-type and SLC35B2-KO cells, respectively. Both FLAG and MYC detection were almost comparable between wild-type and SLC35-KO cells (Fig 5C and 5D), suggesting that FLAG sulfation preferentially occurred in misfolded EGFP-FLAG-CD55.

## Discussion

We performed a genetic screening to identify factors related to the plasma membrane expression of EGFP-FLAG-CD55 (C81A). After enrichment of mutant cells that showed high FLAG staining 3 times, the target genes enriched in mutant cells were determined. *SLC35B2*, encoding PAPS transporter 1, was identified as the gene responsible for the phenotype. The status of tyrosine sulfation by TPST2 changed the surface FLAG staining of cells expressing EGFP-FLAG-CD55 (C81A). However, microscopic analysis revealed that impaired tyrosine sulfation did not induce misfolded GPI-APs expressed from the ER to the plasma membrane. After replacement of the FLAG tag with the MYC tag on misfolded CD55, the increased staining phenomena disappeared. Our results suggest that the FLAG tag on misfolded CD55 is sulfated, reducing its reactivity to antibodies. We proved that the FLAG tag is not a good choice to quantify proteins in secretory pathways.

Tyrosine sulfation is a post-translational modification of secreted and transmembrane proteins that occurs in the trans-Golgi network [4, 5]. Membrane proteins, including receptors, which are vital for a variety of physiological and pathological pathways, are known to be modified with sulfate. GPCRs (G protein-coupled receptors) are one type of well-understood protein bearing this modification [32–34]. Tyrosine sulfation does not change the localization and structure of GPCRs, but lost tyrosine sulfation affects their function, especially their binding with ligands [9, 33, 35]. Previously, some papers showed that tyrosine on FLAG-tags can be modified with sulfate [1, 2]. This modification challenged the possibility of using FLAG-tag for certain experiments, especially targeted protein expression levels.

In addition to the misfolded GPI-APs in this study, it is reported that correctly folded FLAG-tagged proteins such as dopamine receptor 2 expressed in HEK293 cells and neuraminidase expressed in insect cells also affect the anti-FLAG detection [1, 2]. It is not clear why the FLAG-tag on some proteins, but not all, is sulfated. Interestingly, FLAG detection was not affected in EGFP-FLAG-CD55 (WT) cells, suggesting that FLAG modification preferentially occurs in misfolded EGFP-FLAG-CD55 (C81A) cells. Since chaperones, including calnexin, associate with misfolded GPI-APs in the ER and the late secretory pathway [18, 19], the intracellular retention time of misfolded GPI-APs would be longer than that of folded GPI-APs. The prolonged residence time of misfolded EGFP-FLAG-CD55 in the Golgi may increase the possibility of encountering and reacting with TPST2.

There are two PAPS transporters, PAPST1 encoded by *SLC35B2* and PAPST2 encoded by *SLC35B3*, in the human genome. In this study, KO of *SLC35B2* was sufficient for increasing FLAG detection. RNA-seq analysis revealed that the expression of *SLC35B2* (TPM ± SD = 90.4 ± 3.4) was higher than that of *SLC35B3* (TPM ± SD = 18.2 ± 2.1) in HEK293 cells (Huang et al, manuscript under revision). In addition, kinetic analysis showed that PAPST1 has a lower Km value for PAPS than PAPST2 (0.8 μM in PAPST1 and 2.2 μM in PAPST2) [36], indicating that SLC35B2 mainly contributes to the transport of PAPS into the Golgi in HEK293 cells. Besides, two distinct tyrosyl protein sulfotransferases, TPST1 and TPST2, are also encoded in the human genome. TPST1 and TPST2 show distinct pH optima, effects of magnesium supplementation, and substrate specificities [36]. In our study, TPST1 expression only partially restored the phenotypes in TPST1&2-DKO cells, whereas TPST2 strongly suppressed FLAG detection on the cell surface. These results suggest that the FLAG tag on EGFP-FLAG-CD55 (C81A) is sulfated by TPST2.

Mass spectrometric analysis is one of the most powerful tools for the detection of post-translational modifications, including phosphorylation, ubiquitination, glycosylation, methylation, acetylation, and sulfation [37, 38]. Tyrosine sulfation and tyrosine phosphorylation is hard to distinguish by mass spectrometry since they have almost same nominal mass at approximately 80 Da (sulfation: 79.9568 Da; phosphorylation: 79.9663 Da) [39]. However, tyrosine sulfation is less stable than tyrosine phosphorylation according to a study using MS/MS [37], which makes it difficult to detect. Although we tried to detect the sulfate modification on the FLAG-tag in EGFP-FLAG-CD55 (C81A) using mass spectrometry, we could not succeed in the detection. Recently, some anti-sulfotyrosine antibodies have been developed to detect tyrosine sulfation by ELISA, western blotting, and flow cytometry [40–42]. Currently, a commercially available anti-sulfotyrosine monoclonal antibody (called PSG2) was found to bind with sulfotyrosine residues in peptides and proteins independent of their sequence context [40]. The disadvantage is that the PSG2 antibody prefers to recognize sulfotyrosine in the presence of Glu or Asp within ± two residues of tyrosine [40]. To date, there is no direct evidence that the PSG2 antibody recognizes sulfotyrosine on a FLAG tag. It is worth developing a simple and high-throughput method for the detection and measurement of tyrosine sulfation.

## Supporting information

S1 Fig Detection of EGFP-FLAG-CD55 (C81A) using different anti-FLAG antibodies. (PDF)

S1 Table Oligonucleotide primers used in this study. (XLSX)

S2 Table Genotype of knock-out cell lines in this study. (XLSX)
