# Supplementary material for: Sulfation of a FLAG tag mediated by SLC35B2 and TPST2 affects antibody recognition
Source: PLoS One. 2021 May 5;16(5):e0250805. doi: 10.1371/journal.pone.0250805 (PMC8099120; doi:10.1371/journal.pone.0250805)
Supplement: S1 Fig — (PDF) [file pone.0250805.s001.pdf]

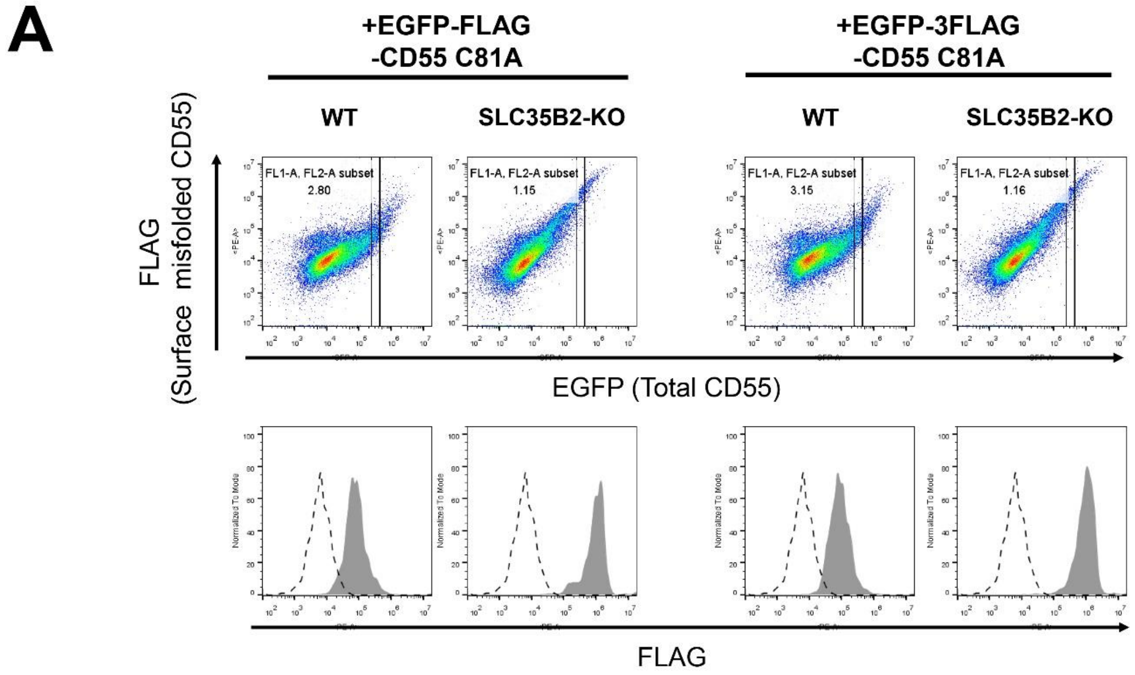

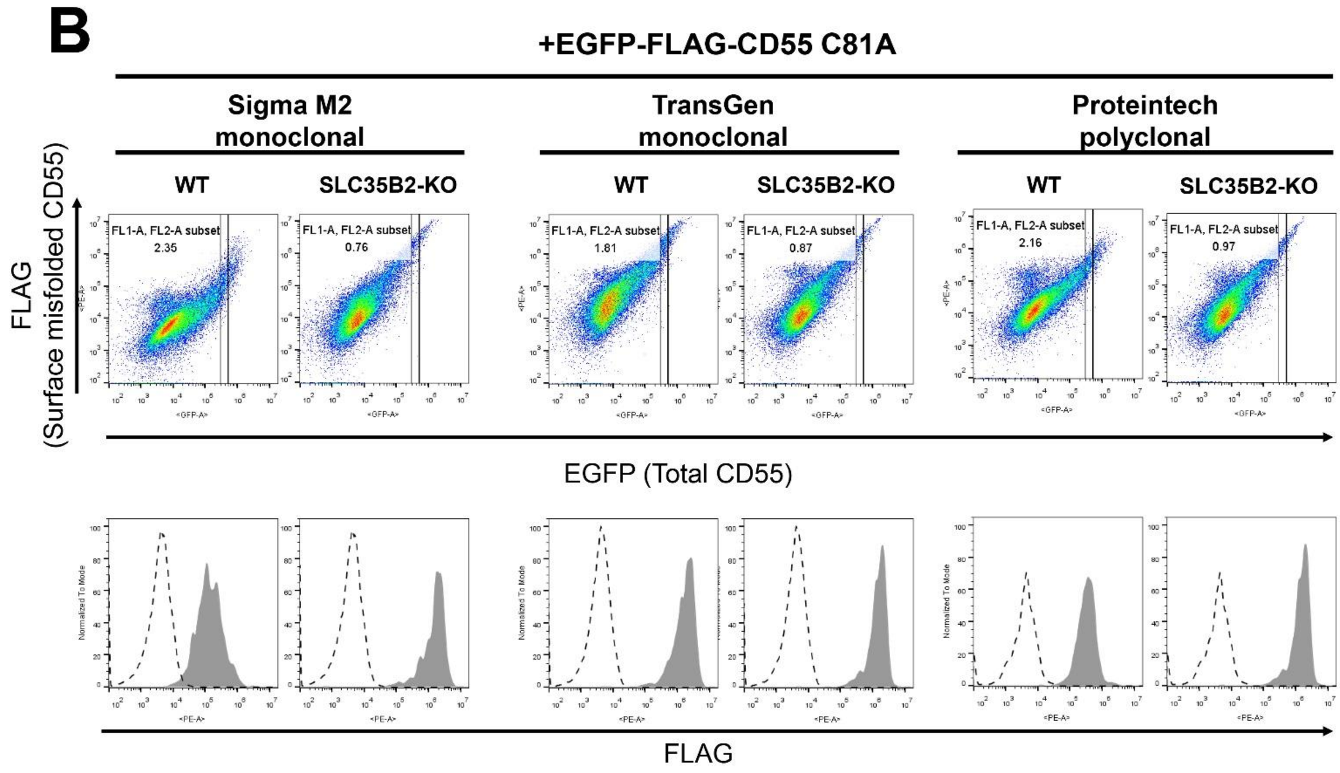

S1 Fig. Detection of EGFP-FLAG-CD55 (C81A) using different anti-FLAG antibodies

A. EGFP-FLAG-CD55 (C81A) or EGFP-3FLAG-CD55 (C81A) was transiently transfected into HEK293WT and SLC35B2-KO cells, respectively. Three days after transfection, cells were harvested and analyzed with anti-FLAG antibody (Sigma M2). The GFP-positive regions were gated, and cell surface staining of anti-FLAG were analyzed by flow cytometry. The results are representative of at least two independent experiments.

B. HEK293WT and SLC35B2-KO were transiently transfected with EGFP-FLAG-CD55 (C81A). Three days after transfection, cells were harvested and analyzed with different anti-FLAG antibodies (from left to right: Sigma M2, TransGen HT201-01, Proteintech 20543-1-AP). The GFP-positive regions were gated, and cell surface staining of anti-FLAG were analyzed by flow cytometry. The results are representative of at least two independent experiments.
